# P072. The visual cortical excitability in pediatric migraine as tested by sound-induced flash illusions

**DOI:** 10.1186/1129-2377-16-S1-A75

**Published:** 2015-09-28

**Authors:** Salvatore Di Marco, Giuseppe Cosentino, Laura Pilati, Roberta Baschi, Simona Maccora, Maria Aprile, Filippo Brighina, Brigida Fierro

**Affiliations:** Department of Experimental Biomedicine and Clinical Neuroscience (BioNeC), University of Palermo, Palermo, Sicily

## Objectives

Sound-induced flash illusions (SIFI) depend on visual cortex (V1) excitability[1]. In adults with migraine, in response to visual-acoustic illusions, V1 is hyperexcitable[2]. Susceptibility to SIFI is increased in children than adults. During childhood there is a change in sensory dominance: acoustic dominant switching to a visual[3]. Here we used SIFI to evaluate V1 excitability in children with migraine assessing also age-related differences in cross-modal audio-visual perception.

## Materials

Twelve children (7 females) affected by migraine without aura: mean age: 10.17±2.76 years, disease duration: 2.91±2.34 years and frequency of attacks: 4.17±3.76/months.

Fifteen healthy children (11 females), mean age 10.61±2.92 years and twenty-four healthy adults (12 females), mean age 25.12±5.74 years with no familiarity for migraine. All subjects were not taking any drugs known to affect cortical excitability. Migraineurs were examined interictally.

## Methods

Visual (flash) and sound (beep) stimuli were presented with different combinations: multiple flash trials where a single beep caused the perception of less flashes, “fusion illusions” and trial where multiple beeps with single flash, induced perception of more flashes, “fission illusion”. Each combination was randomly presented 10 times. At the end of each presentation the subject had to indicate the number of the flashes seen.

## Results

Children saw more illusions than adults (fusions p < .005, fissions p < .00001). Children with migraine did not differ from age matched controls in the illusory percept of fission or fusion, but they perceived more flashes (p < .05) in multiple flash trials with or without beep.

## Conclusions

The increased number of SIFI seen by children is likely due to the higher propensity of visual stimulation driven by auditory stimulus, probably because of acoustic dominance typical for the age. Even if no differences in fission or fusion illusory percept between controls and patients emerged, the increased ability of migraine children to perceive flashes, even outside migraine attack, reveals a hyper-functional visual cortex in migraine also in pediatric age. The sound-induced flash illusions proved to be a valid tool for testing the visual cortical responsivity in pediatric migraine.

Written informed consent to publication was obtained from the patient(s).
